# A Randomized Double-Blinded Dose-dependent Study of Metaraminol for Preventing Spinal-Induced Hypotension in Caesarean Delivery

**DOI:** 10.3389/fphar.2021.608198

**Published:** 2021-05-12

**Authors:** Fei Xiao, Wen-Ping Xu, Han-Qing Yao, Jia-Ming Fan, Xin-Zhong Chen

**Affiliations:** ^1^ Department of Anesthesia, Women’s Hospital, Zhejiang University School of Medicine, Hangzhou, China; ^2^ Department of Anesthesia, Jiaxing University Affiliated Women and Children Hospital, Jiaxing, China

**Keywords:** metaraminol, hypotension, Spinal, anesthesia, cesarean delivery

## Abstract

**Purpose:** Prophylactic infusion of a vasopressor is preferred as a rational choice in clinical practice in Cesarean delivery. Metaraminol is one of most common vasopressors used in obstetric clinical practice. However, the dose-response of metaraminol has not been fully determined and the optimal infusion dose is unknown. Therefore, this study aimed to determine the median effective dose (ED50) and 90% effective dose (ED90) of weight-based fixed-rate metaraminol infusions for preventing spinal-anesthesia-induced hypotension in patients having combined spinal-epidural anesthesia for elective Caesarean delivery.

**Methods:** One hundred and seventeen patients with singleton pregnancies were enrolled and randomly allocated into one of five groups in this study. Patients received prophylactic metaraminol infusion at a fixed rate of 0, 0.25, 1.0, 1.75 or 2.5 μg/kg/min in each group immediately after induction with intrathecal 10 mg of hyperbaric bupivacaine mixed with 5 μg of sufentanil. An effective prophylactic dose was defined as no occurrence of hypotension during the period of spinal introduction and neonatal delivery. Values for ED50 and ED90 of prophylactic infusion of metaraminol were calculated using probit regression. Characteristics of spinal anesthesia and side effects were recorded.

**Results:** The ED50 and ED90 values of weight-based fixed rate of metaraminol infusion were 0.64 (95% CI, 0.04–1.00) μg/kg/min and 2.00 (95% CI, 1.58–2.95) μg/kg/min respectively. The incidence of hypotension decreased with an increased infusion rate of metaraminol in the five groups (test for trend, *p* < 0.001). The incidence of hypotension was similar between group 0 and 0.25, but significant higher than other groups; the incidence of hypotension was also similar between group 1.0 and 1.75, but higher than group 2.5. The incidence of reactive hypertension was significantly higher in group 2.5 compared to the other groups. Physician interventions were more frequent in group 0, 0.25 and 2.5 than in group 1.0 and 1.75 (adjusted *p* < 0.001). No difference was found in neonatal outcomes, including Apgar score and pH value of the umbilical artery.

**Conclusion:** In summary, we have compared four different prophylactic weight-based infusion doses of metaraminol for preventing post-spinal hypotension in Cesarean delivery. The ED_50_ and ED_90_ values of metaraminol infusion for preventing spinal anesthesia-induced hypotension were 0.64 μg/kg/min and 2.00 μg/kg/min, respectively. This finding may be helpful for guiding clinical practice and further research.

## Introduction

Spinal anesthesia is widely used in Cesarean delivery because of its rapid onset and effectiveness (Gizzo et al., 2014). Maternal hypotension frequently occurs during spinal anesthesia and is associated with undesirable maternal and fetal outcomes (Ngan Kee, 2010; Fitzgerald et al., 2020). Various strategies for preventing maternal hypotension have been suggested by clinical anesthesiologists; however, prophylactic infusion of a vasopressor seems to be more attractive in clinical obstetric practice (Ngan Kee et al., 2004; Fitzgerald et al., 2020).

Studies focused on phenylephrine have shown that it can replace ephedrine for the prevention or treatment of spinal-induced hypotension, due to less neonatal acidosis (Mercier et al., 2001; Ngan Kee et al., 2008; Magalhães et al., 2009). However, reactive bradycardia and lower cardiac output may accompany high doses of phenylephrine (Ngan Kee, 2010; Stewart et al., 2010; Fitzgerald et al., 2020). Many studies have described norepinephrine and metaraminol as effective alternatives to phenylephrine for the prevention and treatment of hypotension during spinal anesthesia for Cesarean delivery (Ngan Kee et al., 2015; McDonnell et al., 2017; Chao et al., 2019; Singh et al., 2020). Several dose-response studies have investigated and determined the ideal dose of norepinephrine infusion for the management of post-spinal anesthesia hypotension (Hasanin et al., 2019; Fu et al., 2020; Wei et al., 2020).

Although prophylactic infusion of a vasopressor is preferred as a rational choice in clinical practice of cesarean delivery, the dose-response of metaraminol has not been fully determined and the optimal infusion dose is unknown. The information in this context is extremely important for guiding clinical practice. The primary aim of this study was to determine the dose-response characteristics of weight-based fixed-rate metaraminol infusions for preventing spinal-anesthesia-induced hypotension in patients having combined spinal-epidural anesthesia for elective Caesarean delivery. The secondary aim was to evaluate maternal hemodynamics, neonatal outcomes and side effects.

## Materials and Methods

### Study Design

This study was approved by the Ethical Review Board in Jiaxing University Affiliated Women and Children Hospital, Jiaxing, China (no. 20200007). The trial was registered before patient enrollment at chictr. org.cn (ChiCTR2000029490, Principal investigator: Fei Xiao, Date of registration:February 2, 2020). All patients provided written informed consent. This study commenced on February 17, 2020 and was completed on April 21, 2020.

One hundred and seventeen full-term women aged 22–40 years old with singleton pregnancies scheduled for elective Cesarean delivery were recruited for this study. Exclusion criteria were preeclampsia or hypertension, pre-existing or gestational diabetes, body mass index (BMI) > 35 kg/m^2^, any contraindications to regional anesthesia and cases with a sensory plane block that did not reach T6 or higher.

The study design was a double-blinded, prospective, randomized controlled trial (RCT). Patients were allocated into one of five groups using a random-number sheet, which was generated by computer software (Microsoft Excel, Redmond, Washington). The random-number sheet was placed into a sealed, non-transparent envelope, which was opened after initiation of patient enrollment. The randomization procedure and preparation of the study drug were completed by study investigator, who was not involved in patient management and data collection. In each group, patients received metaraminol infusion at one of five fixed rates (according to our preliminary experiment): 0.25 μg/kg/min (group 0.25), 1.0 μg/kg/min (group 1.0), 1.75 μg/kg/min (group 1.75), 2.5 μg/kg/min (group 2.5) and saline for the control group (group 0). The study drug for each patient was diluted into 50 ml using identical 50-ml syringes, and the solutions were infused at a fixed rate of 50 ml/h. The dose of metaraminol in the 50-ml syringe for each patient in each group varied according to the following formula: 15 (μg/kg) × weights (kg) in group 0.25, 60 (μg/kg) × weights (kg) in group 1.0, 105 (μg/kg) × weights (kg) in group 1.75, 150 (μg/kg) × weights (kg) in group 2.5 and none in group 0. Weights of patients were measured in the morning on the day of surgery.

### Anesthetic Procedure

Patients received no premedication. After arrival in the operating theater, peripheral venous access was secured for each patient via an upper limb vein using an 18 G intravenous cannula, and standard monitoring, including non-invasive blood pressure (NIBP), heart rate, pulse oximetry and electrocardiography, were applied.

Combined spinal-epidural anesthesia was given attheL3–4 interspace via the “needle-through-needle” technique under local anesthesia with patients in the left lateral position. After confirming the presence of clear cerebrospinal fluid (CSF), 10 mg of hyperbaric bupivacaine plus 5 μg of sufentanil was immediately injected intrathecally over 20 s. Before removal of the spinal needle, gentle withdrawal of CSF was ensured to verify the successful administration of the anesthetic; otherwise, the patient was excluded from the study. An epidural catheter was then inserted 3–4 cm into the epidural space. Thereafter, the patient was positioned supine with a wedge placed under the right buttock and 3 L/min of oxygen was delivered by a nasal catheter.

After completion of the intrathecal injection, metaraminol infusion was initiated at the rate of 50 ml/h. Meanwhile, a co-load of 10 ml/kg of warmed lactated Ringer’s solution was infused over 20–30 min and after that, the infusion rate was lowered to keep the vein patent.

### Measurements

The primary outcome of this study is the blood pressure during study period; secondary outcomes of this study include characters of surgical data and sensory block, neonatal outcomes and side effects. The sensory block level was gently checked at 5 min intervals by assessing painful pinprick sensation with the use of an epidural needle (18 G). Surgery was allowed after the sensory block level reached the T6 dermatome, at least. The study period was defined as the interval between intrathecal injection and delivery of the baby. An effective infusion dose was defined as the non-occurrence of hypotension during the study period. On the contrary, the occurrence of hypotension during the study period was defined as an ineffective infusion dose. For each patient, baseline systolic blood pressure (SBP) and heart rate (HR) were determined by calculating the mean of three separate measurements, assessed 3 min apart prior to the initiation of spinal anesthesia. Hypotension was defined as a decrement of 20% of the baseline value of SBP and/or to below 90 mm Hg, and treatment with rapid infusion of lactated Ringer’s solution if SBP >90 mm Hg or administration of 50 μg of phenylephrine if the SBP < 90 mm Hg. Reactive hypertension was defined as an increase in SBP to ≥ 120% of baseline BP values and was managed by stopping the metaraminol infusion. Heart rate less than 50 beats/min was considered as bradycardia and was treated with 0.5 mg of atropine if associated with hypotension; if bradycardia was not accompanied by hypotension, the metaraminol infusion was stopped. To continue or stop metaraminol infusion after neonatal delivery was the decision of anesthesiologists who managed maternal hemodynamics.

Surgery times such as induction-delivery (I–D) interval and side effects, including hypotension, hypertension, bradycardia, nausea and vomiting and shivering were recorded. Physician interventions for treating hypotension, hypertension, bradycardia, stopping or restarting the metaraminol infusion were also recorded.

### Statistical Analysis

The Cochran-Armitage Test for the trend in the incidence of hypotension was utilized to perform sample size calculations using PASS (version 11.0.7; NCSS, LLC, Kaysville, UT). Based on our preliminary data that incidence of hypotension was 75, 60, 40, 25, and 5%,respectivelyfor the five metaraminol infusion doses of 0, 0.25, 1.0, 1.75, and 2.5 μg/kg/min, the total sample size of 40 patients (8 per group) would provide 93% power to detect a linear trend using a two-sided Z test and a significance level of 0.05. To account for possible dropouts, we enrolled 20 patients for each group.

The Kolmogorov-Smirnov test was used to test the normality of continuous data. Normally distributed data such as patients’ demographic data and neonatal outcomes were presented as means (SD) and tested for significance using one-way analysis of variance (ANOVA). The post hoc Bonferroni test was used for pairwise comparisons. Non-normally distributed data, including surgical times and number of interventions, were analyzed using the Kruskal-Wallis test and were presented as medians (interquartile range). The post hoc Dunns test was applied to analyze pairwise comparisons. Categorical data such as the incidence of side effects were analyzed using the Cochran-Armitage chi-squared test for trend; if an overall test of difference among groups was significant, chi-square tests was used for pairwise comparison. The dose-response values for ED50 and ED90 of the metaraminol infusions for preventing post-spinal hypotension were calculated by probit regression analysis. Serial data on SBP in the first 15 min after spinal anesthesia was evaluated by calculating the area under the curve (AUC). The area was calculated for values plotted against time using the trapezium rule and compared by ANOVA; and linear trend analysis was used to test for a linear trend across the groups.

Analyses were performed using IBM SPSS Statistics for Windows version 22.0 (IBM Corp, Armonk, NY) and GraphPad Prism version 5.0 4 (GraphPad Software Inc. San Diego, CA). Where Bonferroni corrections were applied, adjusted *p* values are presented. A *p* value < 0.05 (two-sided) was regarded as statistically significant.

## Results

**FIGURE 1:**
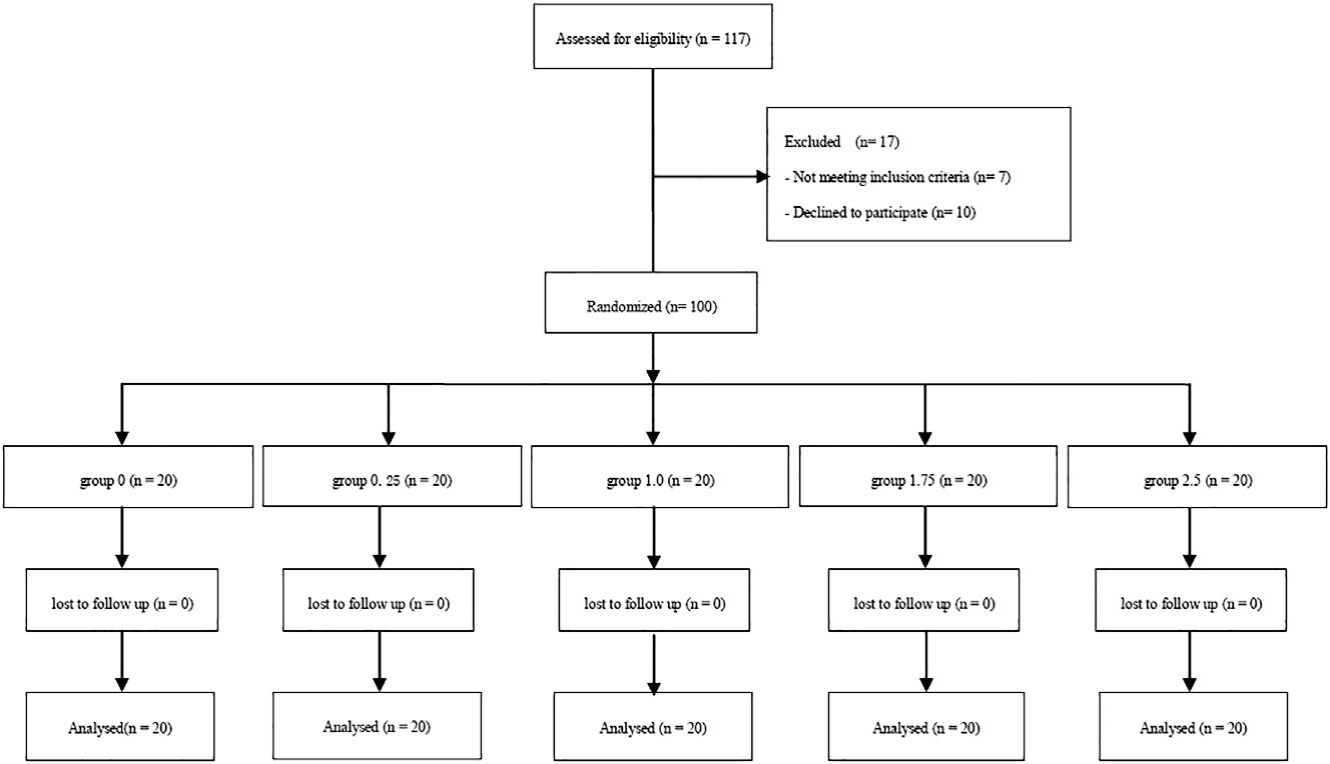
Consort flow diagram.

The Consort Flow is shown in Figure 1. One hundred and seventeen patients were recruited; we excluded seven who did not fulfill the inclusion criteria and 10 who declined to participate in the study. A total of 100 patients were finally enrolled in the study and allocated into one of five groups; these were included in the final analysis. There were no significant differences among groups with regards to demographic data, surgery times and sensory block level (Table 1).

**TABLE 1:** Demographic data, surgical times and sensory block level.

	Group 0 (*n* = 20)	Group 0.25 (*n* = 20)	Group 1.0 (*n* = 20)	Group 1.75 (*n* = 20)	Group 2.5 (*n* = 20)
Age (yr)	29 ± 5	31 ± 4	31 ± 4	32 ± 5	32 ± 5
BMI (kg/m^2^)	27.8 ± 3.0	28.7 ± 3.6	27.9 ± 3.0	27.9 ± 3.4	27.1 ± 3.5
Gestational age (wk)	38 ± 1	39 ± 1	39 ± 1	39 ± 1	38 ± 1
Induction-delivery interval (min)	15.0 (14.0, 16.8)	15.5 (14.0, 19.0)	16.5 (14.3, 19.0)	16.0 (14.3, 18.8)	15.5 (14.3, 17.0)
Sensory block level	T4 (T3-T4)	T4 (T3-T4)	T4 (T3-T4)	T4 (T4-T4)	T4 (T4-T4)

Data are mean ± SD or median (Q).

**FIGURE 2:**
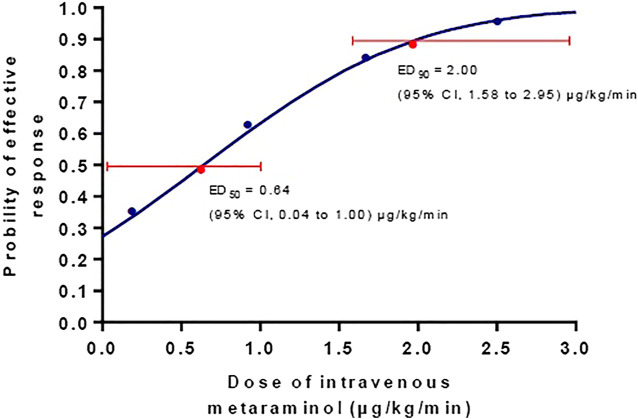
The dose-response curve of metaraminol for preventing spinal anesthesia-induced hypotension. The ED_50_ and ED_90_ values of weight-based fixed rate of metaraminol infusion were0.64 (95% CI, 0.04–1.00) μg/kg/min and 2.00 (95% CI, 1.58–2.95) μg/kg/min respectively.

The incidence of hypotension decreased with increasing infusion rate of metaraminol in the five groups (test for trend, *p* < 0.001) showed in Table 2. The incidence of hypotension was similar between group 0 and 0.25, but significant higher than other groups; the incidence of hypotension was also similar between group 1.0 and 1.75, but higher than group 2.5; and the exact *p* values were presented in Table 2. The dose-response curve of metaraminol for preventing spinal anesthesia-induced hypotension derived from probit analysis is presented in Figure 2. The ED50 and ED90 values of weight-based fixed rate of metaraminol infusion were 0.64 (95% CI, 0.04–1.00) μg/kg/min and 2.00 (95% CI, 1.58–2.95) μg/kg/min respectively.

**TABLE 2:** Hemodynamic changes, side effects and neonatal outcomes.

	Group 0 (*n* = 20)	Group 0.25 (*n* = 20)	Group 1.0 (*n* = 20)	Group 1.75 (*n* = 20)	Group 2.5 (*n* = 20)	*p*-value
Hypotension	16 (80)^a^	13 (65)^b^	6 (30)^c^	5 (25)^d^	0 (0)	<0.0001
Reactive hypertension	0 (0)	1 (5)	0 (0)	1 (5)	9 (45)^e^	<0.0001
Bradycardia	3 (15)	3 (15)	6 (30)	4 (20)	7 (35)	0.132
Nausea or vomiting	9 (45)	6 (30)	3 (15)	4 (20)	3 (15)	0.022
Shivering	5 (25)	4 (20)	4 (20)	5 (25)	4 (20)	0.865
Numbers of physician intervention	2.0 (1.3, 3.0)^f^	2.0 (0, 2.0)^g^	0 (0, 1.8)	0 (0, 0.8)	1.0 (0, 1.0)^h^	0.0002
Apgar score	9 ± 1	9 ± 1	9 ± 1	9 ± 1	9 ± 1	0.685
Umbilical artery pH	7.30 ± 0.05	7.30 ± 0.03	7.30 ± 0.04	7.30 ± 0.05	7.33 ± 0.03	0.08

Data are presented as number (%), median (Q) or mean ± SD. Categorical data were analyzed using the Cochran-Armitage chi-square test for trend. Reactive hypertension was defined as systolic blood pressure >120% of baseline value.

a *p* = 0.003 vs. Group 1.0, *p* = 0.001 vs. Group 1.75, *p* < 0.0001 vs. Group 2.5.

b *p* = 0.025 vs. Group 1.75, *p* < 0.0001 vs. Group 2.5.

c *p* = 0.020 vs. Group 2.5.

d *p* = 0.047 vs. Group 2.5.

e *p* = 0.008 vs. Group 0.25 and 1.75, *p* = 0.001 vs. Group 0 and 1.0.

f Adjusted *p* < 0.0001 vs. Group 1.0 and 1.75.

g Adjusted *p* < 0.0001 vs. Group 1.0 and 1.75.

h Adjusted *p* = 0.006 vs. Group 1.0, *p* = 0.002 vs. Group 1.75.

**FIGURE 3:**
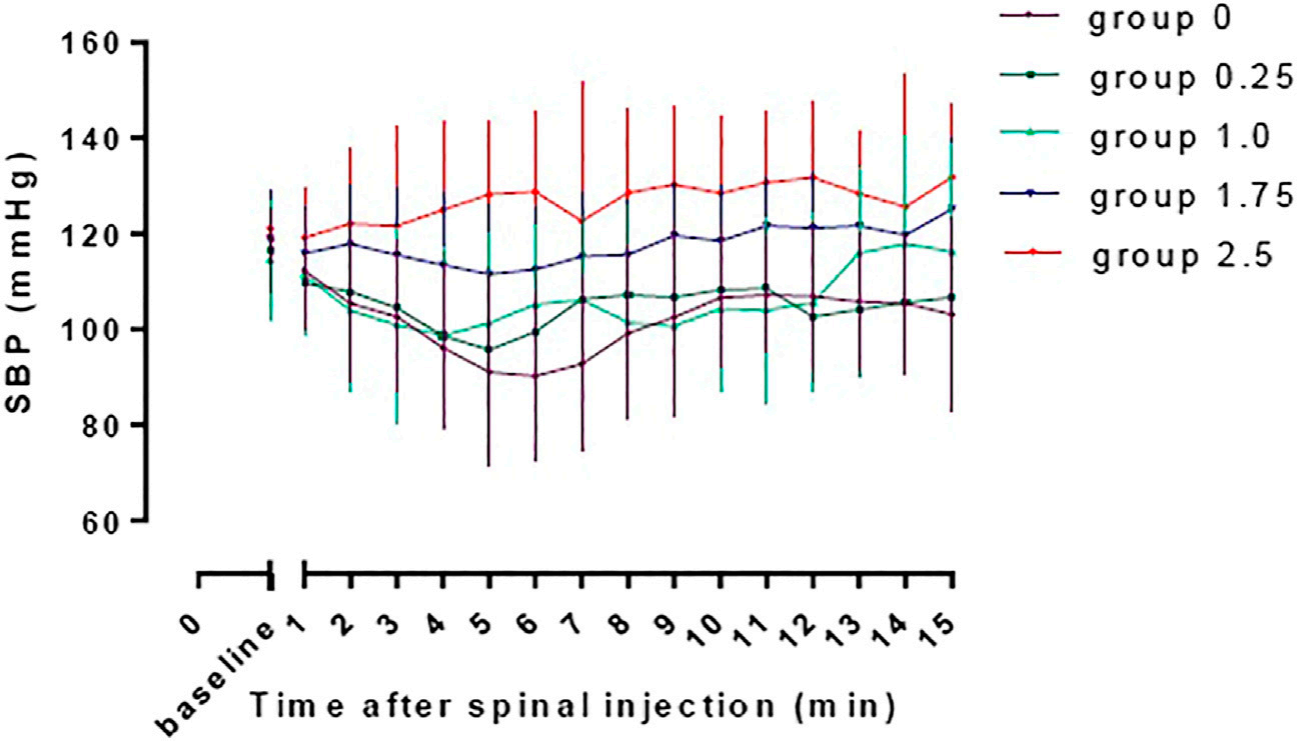
The baseline SBP and SBP in the first 15 min after spinal induction in the five groups. The area under the curve were 2002 ± 51, 2065 ± 41, 2,109 ± 57, 2,355 ± 39, and 2,356 ± 53 min mm Hg in group 0, 0.25, 1.0, 1.75, and 2.5, respectively, and there was a significant linear trend across groups (*p* < 0.001). Significant difference was found in AUC between group 2.5 and group 0, 0.25 and 1.0 (adjusted *p* < 0.001) and between group 1.75 and group 0 and 0.25 (adjusted *p* < 0.001).

The baseline SBP and SBP in the first 15 min after spinal induction in the five groups are shown in Figure 3. The AUC was 2002 ± 51, 2065 ± 41, 2,109 ± 57, 2,355 ± 39 and 2,356 ± 53 min mm Hg in group 0, 0.25, 1.0, 1.75, and 2.5respectively, and there was a significant linear trend across the groups (*p* < 0.001). Significant difference was found in AUC between group 2.5 and group 0, 0.25 and 1.0 (adjusted *p* < 0.001) and between group 1.75 and group 0 and 0.25 (adjusted *p* < 0.001).

Physician interventions were more frequent in group 0, 0.25 and 2.5 than in group 1.0 and 1.75, which are presented in Table 2. There is a significant difference among groups with regards to physician interventions (*p* = 0.0002). Side effects are described in Table 2. Reactive hypertension was significantly higher in group 2.5 compared to the other groups, *p* < 0.0001. The incidence of nausea and vomiting was highest in group 0, and there was a trend for decreasing associated with metaraminol dose (*p* = 0.022), but no difference was found among groups. Neonatal outcomes, including Apgar score and pH value of the umbilical artery were similar among groups.

## Discussion

Results of this randomized prospective dose-response study demonstrate that the ED_50_ and ED_90_ values of weight-based fixed-rate metaraminol infusion are 0.64 (95% CI, 0.04–1.00) μg/kg/min and 2.00 (95% CI, 1.58–2.95) μg/kg/min, respectively for preventing spinal anesthesia-induced hypotension during combined spinal-epidural anesthesia for cesarean delivery. To our knowledge, this is the first study to explore dose-response characteristics of metaraminol for preventing spinal-anesthesia induced hypotension in Caesarean delivery. These findings have the potential to guide initial dose infusions of metaraminol in the prevention of hypotension in obstetric anesthesia.

It is well known that phenylephrine is widely used in obstetric anesthesia, but it is associated with reactive bradycardia and decreased cardiac output (CO), especially when large doses of this vasopressor are administered (Stewart et al., 2010). However, there is international consensus that keeping maternal blood pressure and heart rate (as an alternative measure of CO) at baseline levels are the primary goals of using prophylactic vasopressors (Kinsella et al., 2018). Therefore, metaraminol and norepinephrine, which have weak β-adrenergic receptor agonist activity, are suggested to have the potential to be used as alternatives to phenylephrine during Cesarean delivery, in order to avoid reactive bradycardia (Chao et al., 2019; Singh et al., 2020). In the current study, our findings are consistent with these suggestions; increasing infusion doses of metaraminol effectively reduced the incidence of hypotension without causing reactive bradycardia. However, administration of metaraminol was associated with reactive hypertension (McDonnell et al., 2017; Chao et al., 2019), that is consistent with our study, in which we found the reactive hypertension is dose dependent. Therefore, to determind the dose-response of prophylactic metaraminol is helpful to provide informations of an approprite initial dose for obstetric anesthesia.

In recent times, many studies have described regimens for the administration of metaraminol, which are needed to maintain hemodynamic status during spinal anesthesia in obstetric anesthesia. McDonnell et al. (2017) *.* conducted a randomized double-blinded study to compare metaraminol and phenylephrine infusion for the prevention of hypotension after spinal anesthesia for Cesarean delivery; they reported that 250 μg/min of metaraminol is at least non-inferior to 50 μg/min of phenylephrine with respect to neonatal acid-base outcomes (7.31 vs. 7.28, *p* = 0.0002). And the dose of metaraminol in their study is much higher than that in present study, which may lead to a higher incidence of reactive hypertension in their study (49%). Chao et al. (2019) *.* conducted a meta-analysis and reported that metaraminol may be a more suitable vasopressor than ephedrine and at least no inferior to phenylephrine for spinal anesthesia in cesarean delivery. Moreover, Singh et al. (2020) also suggested that metaraminol and other vasopressors with β-adrenergic receptor agonist activity were less likely to adversely affect the fetal acid-base status. Taken the overall findings together, it can be suggested that metaraminol has the potential to replace phenylephrine as an alternative in obstetric anesthesia; but definitive large-scale studies are needed to explore these questions. Nevertheless, we have provided information on the ideal initial dose of metaraminol infusion for spinal anesthesia in cesarean delivery, which could have benefits for obstetric practice.

We adopted a weight-based infusion based on a prior study that showed less incidence of hypotension with a weight-adjusted regimen of vasopressor than a fixed-dose regimen of vasopressor, for maintaining SBP during spinal anesthesia for cesarean delivery (Mwaura et al., 2016). That the incidence of hypertension was lower than that for other studies may depend on the weight-based infusion strategies and a lower infusion dose in this study. Ngan et al. (Mwaura et al., 2016). described a non-weight adjusted system using BP as a negative feedback to manage the infusion rate, and this appeared to be sensitive in keeping SBP close to baseline; but this system is not yet available for commercial use. Titration of the infusion rate could be more suitable for hemodynamic adjustment than a fixed-dose. However, there is limited evidence showing which strategy (a fixed rate or variable titration rate) is better for clinical practice. Further studies that focus on this issue are warranted.

The ultimate goal of management with vasopressors for Cesarean delivery under spinal anesthesia is to minimize the maternal side effects and to improve neonatal outcomes, such as the acid-base status. In this study, with an increasing dose of metaraminol, the incidence of hypotension and nausea and vomiting was decreased accordingly. The incidence of hypertension was increased for the 2.5 μg/kg/min infusion, although hypertension was quickly treated by stopping the infusion. In theory, a higher level of BP would benefit uterine-placenta perfusion, which mainly depends on SBP and placental vascular resistance. Interestingly, there were no significantly differences in neonatal outcomes among the groups. Nevertheless, this study did not have sufficient power to evaluate these findings.

Limitations of this study deserve consideration. First, we evaluated hemodynamic status using only SBP and HR without assessing CO and SVR, which may reflect hemodynamic status more precisely; although SBP and HR are regarded proxies for CO. Second, all the patients recruited in this study were scheduled for elective Cesarean delivery and did not have any abnormal maternal or fetal medical conditions; hence the results of this study are limited in generalizability, particularly for patients undergoing emergency Cesarean delivery and/or those with maternal or fetal medical conditions.

In summary, we have compared four different prophylactic weight-based infusion doses of metaraminol for preventing post-spinal hypotension in Cesarean delivery. The ED50 and ED90 values of metaraminol infusion for preventing spinal anesthesia-induced hypotension were 0.64 μg/kg/min and 2.00 μg/kg/min, respectively. This finding may be helpful for guiding clinical practice and further research.

## Data Availability

The original contributions presented in the study are included in the article/Supplementary Material, further inquiries can be directed to the corresponding author.
